# Phaeochromocytoma Mimicking Scleroderma

**DOI:** 10.1155/2011/917453

**Published:** 2011-02-23

**Authors:** Joan Joo-Ching Khoo, Edward John Pratt

**Affiliations:** Department of Medicine, Changi General Hospital, 2 Simei Street 3, Singapore 529889

## Abstract

We describe a patient with adrenal phaeochromocytoma who presented with peripheral vasospasm and ischaemia and other manifestations mimicking features of systemic sclerosis, which resolved after resection of the tumor. Phaeochromocytoma should be suspected in patients with features of scleroderma who are negative for auto-antibodies.

## 1. Introduction

Phaeochromocytomas are catecholamine-secreting tumours of chromaffin cells in the adrenal gland or in extra-adrenal sympathetic ganglia (where they are called paragangliomas), which are frequently sought when a hypertensive patient presents with the “classical” triad of headache, palpitations, and diaphoresis. However, the diagnosis may be overlooked when it presents with atypical symptoms. Timely diagnosis is important as hypertension is curable by tumour resection. We describe the case of a middle-aged female with incidentally diagnosed phaeochromocytoma, who presented with symptoms suggesting systemic sclerosis.

## 2. Case Report

A 45-year-old Chinese female presented to the rheumatology outpatient clinic with a 2-month history of daily episodes of spontaneous blanching of the fingers of both hands, associated with numbness, which resolved within a few minutes. She also noticed tightening of the skin over her fingers and increased nail fragility but denied fever, rashes, joint pains, and changes in weight, appetite, and bowel habits. Physical examination revealed sclerodactyly, onycholysis, and small pits on the index and middle fingers of the right hand, leading to a diagnosis of scleroderma. Her peripheral pulses were of normal volume, capillary refill time was normal, and no discolouration was noted. She gave a history of oesophageal reflux and difficulty in swallowing, which were partially relieved by omeprazole. Her past medical history was significant for hypertension diagnosed six months ago. She had no family history of autoimmune disease. Blood pressure (BP) in the clinic was 160/95 mmHg on atenolol 50 mg om and amlodipine 10 mg om. Hemoglobin level, renal and thyroid function tests were normal. She tested negative for antinuclear antibody, anti-double-stranded DNA, anti-Ro, anti-La, anti-RNP, anti-Sm, anti-Jo-1, and Sc1-70 antibodies.

**Figure 1:**
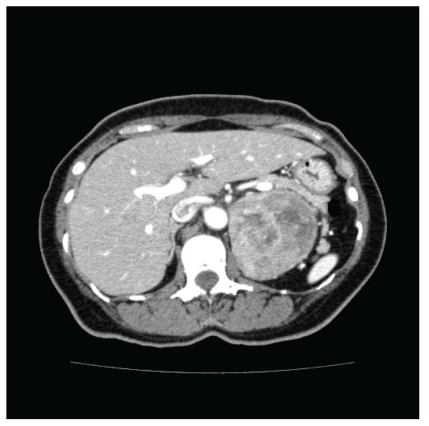
Left adrenal phaeochromocytoma with areas of necrosis and hemorrhage.

Computed tomography (CT) thorax, performed to evaluate pulmonary complications of systemic sclerosis, showed no pulmonary fibrosis or embolism but incidentally detected a heterogeneous left adrenal mass 7.5 × 7 cm in diameter with areas of necrosis and hemorrhage and enhancement with contrast (Figure 1). The diagnosis of adrenal phaeochromocytoma was confirmed by elevated 24-hour urinary catecholamine and metanephrine levels. She denied a history of headache, palpitations, or diaphoresis. There was no family history of phaeochromocytoma.

Good BP control (110–130/60–80 mmHg with pulse rate 60–80/minute) was achieved with phenoxybenzamine and atenolol before an uncomplicated adrenalectomy. Histology of the adrenal tumor was consistent with a phaeochromocytoma. Postoperative urine catecholamine and metanephrine levels were normal. Three months after surgery, her BP was 120/60 mmHg without any medication. The symptoms and signs of “systemic sclerosis” (blanching of hands, sclerodactyly, finger pitting, reflux, and dysphagia) had completely resolved.

## 3. Discussion

This patient had an adrenal phaeochromocytoma presenting as systemic sclerosis, with complete resolution of peripheral ischaemia and oesophageal dysmotility after removal of the tumor. Although our patient had symptoms suggesting Raynaud's phenomenon and fulfilled two (sclerodactyly and digital pulp atrophy as evidenced by pitting) of the three minor criteria, with 97% sensitivity and 98% specificity for the diagnosis of systemic sclerosis [1], she tested negative for auto-antibodies. More than 90% of patients with systemic sclerosis are positive for antinuclear antibodies [2], suggesting that our patient's symptoms were likely to have been caused by sympathetic overactivity due to catecholamine excess rather than autoimmune disease, as they resolved after resection of her adrenal phaeochromocytoma and normalization of catecholamine levels. Sympathetic overactivity associated with oesophageal dysmotility [3] could have accounted for our patient's symptoms of reflux and dysphagia. In patients with systemic sclerosis and mixed connective tissue disease, oesophageal motor dysfunction was associated with elevated plasma titres of anti-Sc170 antibodies and cardiovascular autonomic neuropathy [4]. It was therefore unusual that our patient was autoantibody negative, suggesting that her symptoms of oesophageal dysmotility were not due to systemic sclerosis or connective tissue disease. 

Vasospasm, with or without the full spectrum of colour changes of Raynaud's phenomenon, is associated with peripheral arterial digital ischaemia. Phaeochromocytoma rarely presents with acute limb ischaemia [5, 6]. Significant negative correlations between plasma catecholamine concentration and dermal blood flow have been demonstrated in patients with phaeochromocytomas [7]. Hence, chronic recurrent arterial vasospasm, due to excessive catecholamine production from phaeochromocytoma, was likely to have caused scleroderma-like features of our patient's hands, with blanching due to surges of catecholamine production and sustained elevation leading to pulp atrophy and skin tightening. Phaeochromocytoma-induced hypertension and peripheral ischaemia may be exacerbated by the administration of atenolol, due to vasoconstriction induced by unopposed alpha-adrenergic effect after beta-adrenergic blockade. Our patient only achieved optimal BP control when the alpha-blocker phenoxybenzamine was substituted for amlodipine. Hypertension was cured by removal of the tumor, emphasizing the importance of identifying secondary causes of uncontrolled hypertension of recent onset. 

## 4. Learning Point

Investigation for phaeochromocytoma is advisable in patients with suspected scleroderma and negative auto-antibodies, especially with a history of hypertension.
